# Influence of the Si-Layer Thickness on the Structural, Compositional and Resistive Switching Properties of SiO_2_/Si/SiO_2_ Stack Layers for Resistive Switching Memories

**DOI:** 10.3390/ma18245539

**Published:** 2025-12-10

**Authors:** Alfredo Morales-Sánchez, Karla E. González-Flores, Jesús M. Germán-Martínez, Braulio Palacios-Márquez, Juan F. Ramírez-Rios, Javier Flores-Méndez, Alfredo Benítez-Lara, Juan R. Ramos-Serrano, Luis Hernández-Martínez, Mario Moreno-Moreno

**Affiliations:** 1 Electronics Department, Instituto Nacional de Astrofísica, Óptica y Electrónica, Puebla 72840, Mexico; karla.gonzaflores@gmail.com (K.E.G.-F.); jemigema@inaoep.mx (J.M.G.-M.); bpalacios@inaoep.mx (B.P.-M.); juanfedericoramirezrios@gmail.com (J.F.R.-R.); abenitezl@inaoep.mx (A.B.-L.); juan.ramos@inaoep.mx (J.R.R.-S.); luish@inaoep.mx (L.H.-M.); mmoreno@inaoep.mx (M.M.-M.); 2 Área de Ingeniería, Benemérita Universidad Autónoma de Puebla, Ciudad Universitaria, Blvd. Valsequillo y Esquina Av. San Claudio s/n, Col. San Manuel, Puebla 72570, Mexico; javier.floresme@correo.buap.mx; 3 Tecnológico Nacional de México/I.T. Puebla-Mecánica, Av. Tecnológico No. 420, Maravillas, Puebla 72220, Mexico

**Keywords:** silicon nanocrystals, resistive switching memories, SiO_2_/Si multilayers

## Abstract

This work focuses on developing resistive switching (RS) devices using thermally annealed (TA) SiO_2_/Si multilayers (ML). Three SiO_2_/Si bilayers were deposited with an additional 10 nm SiO_2_ layer as a dielectric barrier layer on top of the ML. The SiO_2_ layers were 6 nm thick, while the thickness of the Si layers varied from 2, 4, and 6 nm, and were labeled as ML-62, ML-64, and ML-66, respectively. X-ray photoelectron spectroscopy analysis revealed well-defined ML structures before TA. However, after TA, samples ML-64 and ML-62 showed discontinuities due to diffusion between neighboring Si layers, increasing the dimensions of the Si-rich regions. In fact, the concentration of elemental Si (Si^0^) within the intermediate Si layer increases as the Si layer becomes thinner. Consequently, the size of Si-nanocrystals, created after TA, increases from 6 to 8.5 nm for ML-66 to ML-62, as confirmed by Raman and transmission electron microscopy analysis. The composition discontinuities and loss of the ML structure resulted in erratic electrical behavior, with an electroforming (EF) voltage as high as −14 V in sample ML-62. For the ML-66, which retained the ML structure, the EF voltage was reduced to −4 V, showing SET/RESET values of around ±3 V and stable electrical behavior, with an ON/OFF ratio of up to seven orders of magnitude. This demonstrates the importance of the ML design in the operation of RS devices.

## 1. Introduction

The memristor, based on the resistive switching (RS) effect, has received much attention due to multilevel conductance, high switching speed, and low energy consumption, making it a candidate for developing systems that emulate the artificial synapse [1,2,3,4,5,6,7,8]. The memristor exhibits a RS behavior from a high-resistance state (HRS) to a low-resistance state (LRS) for the SET (ON) process and RS from LRS to HRS for the RESET (OFF) process when it is electrically biased. These SET and RESET processes can be obtained at the same voltage polarity (unipolar RS) or with opposite polarities (bipolar RS) [6,9,10]. The unipolar RS is typically observed in binary transition metal oxides (TMOs) like TiO_2_, NiO, CuO, HfO_2_, and Ta_2_O_5_ [11,12,13,14,15] while the bipolar RS has been reported for complex oxides such as Pr_0.7_Ca_0.3_MnO_3_ (PCMO) and SrTiO_3_ [16,17,18], but also in binary oxides out of stoichiometry like TiO_2−x_, HfO_2−x_, and Ta_2_O_5−x_ [13,14]. In recent years, the study of the RS phenomenon has been extended to nanocomposites such as Au-TiO_2_, Ba:HfO_x_, ZnO/NiO, Cu/(Co-FeB)_x_(SiO_z_)_100−x_/a-LiNbO_y_/Cu/Cr, and SrTiO_3_-CeO_2_, demonstrating interest in studying this phenomenon for its implementation in electronic devices [19,20,21,22,23].

The RS phenomenon has been related to the formation and annihilation of conductive filaments (CF) through electrochemical or electro-thermal (Joule heating) processes [24,25]. Although CFs could overcome the miniaturization challenges of the flash memory [26,27], their stochastic formation produces variability in the memristor performance [28] and, as a result, a significant degradation in the RS process [29]. Therefore, the control of the CF formation is essential for its application in RS memories. However, the formation of CFs at the nanoscale and their specific place within the active material is still a matter of research.

Silicon-rich dielectric materials, including SiO_x_, SiN_x_, and SiC_x_, are some other active materials that have also shown RS properties [30,31,32,33]. These active materials are important since they are compatible with the complementary metal-oxide-semiconductor (CMOS) technology, allowing for Si-based monolithic integration. The RS process in these materials has been related to the formation/annihilation of CFs created by Si-nanocrystals (Si-ncs) embedded in the dielectric matrix [34,35,36]. In fact, an in situ TEM study revealed the formation/annihilation of the CF formed by crystallization/amorphization of a Si path [37]. However, at present, the complete integration of these materials presents major challenges, such as high thermal annealing temperatures that allow the formation of Si-ncs in dielectric matrices, which are key components in the operation of Si-based devices. Several efforts have been developed to improve the RS properties in this kind of material. In 2017, Kwon S et al. proposed a conical nanoporous structure to confine Si-ncs-based CFs to obtain fast and scalable RS memories [38]. Y T Chen covered the SiO_2_ film with a Si thin film to obtain a more robust and uniform CF [39]. The use of Si-ncs in multilayer (ML) structures has also been proven to show the RS phenomenon [40,41,42]. Moreover, additional RS levels have been obtained with the use of Si-ncs through light excitation or the use of MLs with several Si-ncs layers in the ML [41,42,43,44].

This work is focused on the study of structural, compositional, and electrical properties of Si-ncs embedded in SiO_2_/Si ML-based MOS-like devices. The study focuses on the effect of the Si-layer thickness in SiO_2_/Si MLs. Changes in the RS properties are correlated with the compositional and structural properties of the Si-ncs/SiO_2_ MLs.

## 2. Materials and Methods

The SiO_2_/Si ML structures were deposited with an RF magnetron sputtering system in an argon plasma at 500 °C. Three SiO_2_/Si bilayers were deposited with an additional 10 nm-thick SiO_2_ layer as a dielectric barrier layer on top of the ML. The thickness of SiO_2_ layers was fixed at 6 nm, while the thickness of the Si layers varied from 2, 4, and 6 nm, as listed in Table 1. Before ML deposition, the Si substrates were cleaned using an ultrasonic bath with acetone, ethanol, and deionized water, followed by submersion in an aqueous solution of hydrofluoric acid (HF) to etch the native oxide. All SiO_2_/Si MLs were thermally annealed (TA) at 1100 °C for 1 h under N_2_ atmosphere in a quartz tube furnace to promote the formation of Si-ncs in the Si layers. The atomic composition in-depth profile was analyzed by X-ray photoelectron spectroscopy (XPS) using a Thermo Scientific Escalab 250Xi XPS system (ThermoFisher, Waltham, MA, USA) equipped with an Al-Kα (1486.7 eV) monochromatic X-ray source. The formation of Si-ncs within the SiO_2_/Si MLs was analyzed by Raman spectroscopy using a Horiba Jobin-Yvon LabRam spectrometer (Horiba Jobin Yvon GmbH, Kyoto, Japan) (excitation, λ = 532.1 nm) and corroborated by transmission electron microscopy (TEM, JEOLTM, JEM-2200, and JEM-F200).

For the electrical characterization, MOS-like devices were fabricated as follows: an Al electrode (300 nm thick) was deposited over the SiO_2_ barrier layer by electron beam evaporation (e-beam), which acts as the top electrode (TE). The gate was defined by standard photolithography and lift-off processes with square patterns, with an area of 6.25 × 10^−4^ cm^2^. A ~300 nm thick Al layer was also deposited on the back side of the Si substrates by e-beam evaporation as the bottom electrode (BE). Finally, the MOS-like devices were thermally annealed at 460 °C, forming gas for 20 min to improve the ohmic contact. The current–voltage (I–V) characteristics were measured under ambient conditions using a Keithley 4200-semiconductor characterization system using a probe station. Voltage bias was applied to the Al-TE while the Si substrate was grounded.

## 3. Results

**Figure 1:**
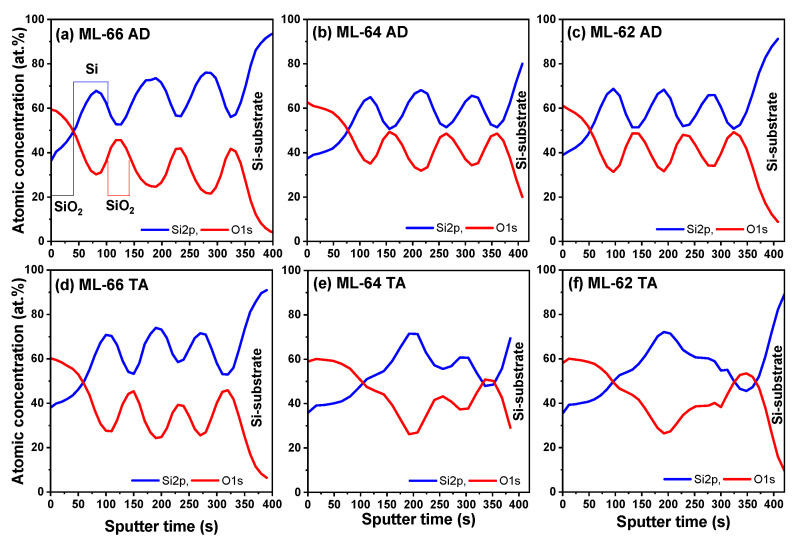
XPS depth profile of AD and TA SiO_2_/Si ML. (**a**,**d**) correspond to ML-66, (**b**,**e**) to ML-64, and (**c**,**f**) to ML-62.

The in-depth composition profile of the different SiO_2_/Si ML structures is shown in Figure 1. The Si2p and O1s signals were measured at different depths, which were achieved by etching the sample surface with Ar ions. The atomic percentage of the Si2p and O1s profiles has several maxima and minima as a function of erosion time, which confirms the ML system. In fact, in as-deposited (AD) MLs (without thermal annealing), Figure 1a–c, the number of maxima for Si2p and O1s signals is completely related to the number of (3) bilayers, with different maxima of Si and O profiles. The typical diffusion of Si atoms from the Si layers towards the neighboring SiO_2_ layers already reported before is observed, obtaining SiO_x_ (x < 2) layers rather than SiO_2_ in the ML system [45].

After the TA process (Figure 1d–f), the three different maxima and minima of the Si2p and O1s profiles are only preserved for the ML-66, which contains the thicker Si layer. As the Si layer thickness decreases (ML-64 and ML-62), the excess Si in the first and third Si layers strongly reduces (Figure 1e,f). Furthermore, the SiO_2_ layers located between the first Si layer and the Si substrate are characterized by a higher degree of silicon excess compared to the upper SiO_2_ layer. The high silicon content in the SiO_x_ layers, where SiO_2_ was nominally deposited, does not prevent the diffusion of excess silicon from the neighboring Si layers. In fact, as reported by [42], the diffusion coefficient of Si in SiO_2_ nanolayers depends on their thickness; thus, the diffusion coefficient is higher for thinner films.

**Figure 2:**
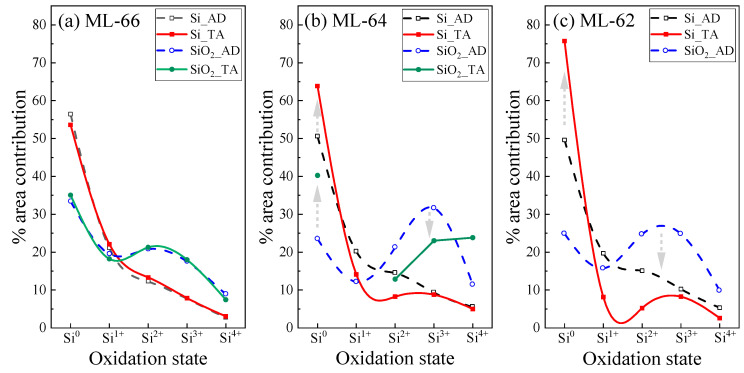
Contribution by area percentage of the different oxidation states for as-deposited and thermally annealed MLs: (**a**) ML-66, (**b**) ML-64, and (**c**) ML-62. Arrows indicate compositional changes.

It is well known that the composition of a SiO_x_ film can be explained by the random bonding model [46,47,48], where different Si oxidation states can be analyzed by the Si2p-XPS spectrum. Five different tetrahedral units, Si–(Si_4−n_–O_n_) with *n* = 0–4, that correspond to Si^0^, Si^1+^, Si^2+^, Si^3+^, and Si^4+^ can be obtained. Figure 2 shows the data obtained by a deconvolution process of the Si2p-XPS spectra in the middle region of the multilayer (second bilayer), taking as reference the maximum atomic percentage of Si and O for the Si and SiO_2_ layer, respectively. This analysis was performed for the AD and TA samples to study the diffusion effect in the ML structures. The Si2p-XPS spectra presented 5 main bands located at 99.3 ± 0.2 eV, 100.3 ± 0.2 eV, 101.5 ± 0.3 eV, 102.6 ± 0.2 eV, and 103.6 ± 0.2 eV, which are related to elemental silicon (Si^0^), Si^1+^, Si^2+^, Si^3+^, and Si^4+^, respectively. As observed in Figure 1a–c, the AD-MLs present a well-defined structure, where the regions corresponding to the Si and SiO_2_ layers can be easily identified. However, when subjected to TA, the ML-64 and ML-62 present significant changes compared to the original structures, with ML-66 being the only one with a stable behavior (Figure 1d–f).

For the ML-66, Figure 2a, the highest contribution in the Si and SiO_2_ layers is generated by the Si^0^ with values of approximately 55.0 and 34.3%, respectively. But most importantly, there are no significant changes in the oxidation states between AD-ML and TA-ML. On the contrary, for ML-64 and ML-62 (Figure 2b,c), it is possible to observe an increase in the Si^0^ contribution after TA, as indicated by the gray arrows. For ML-64, it increases up to 13.3 and 16.5% in the Si and SiO_2_ layers, respectively; while an increase of 26.1% is obtained in the Si layer for ML-62, in which the total loss of the ML structure was observed after TA (Figure 1f). Due to this phenomenon, Figure 2c only shows the oxidation states of the Si layer (Si_TA), as it is considered a single film without a defined SiO_2_ region. Similarly, a reduction in the Si^1+^ to Si^4+^ oxidation states is observed in these MLs. This behavior would be related to the fact that a higher concentration of Si alters the distribution of this element in the dielectric matrix and its incorporation in the Si–(Si_4−n_–O_n_) structure, with *n* = 1–4, such that the higher the presence of Si, the oxidation states would suffer a reduction, as observed in Figure 2b,c.

**Figure 3:**
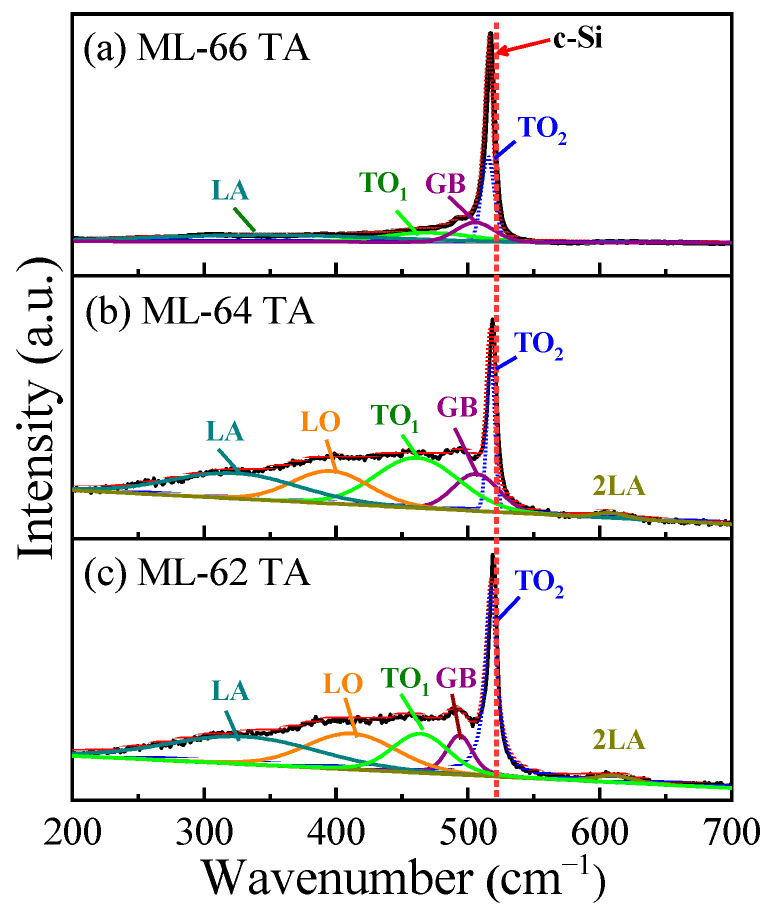
Raman spectra of thermally annealed SiO_2_/Si MLs with the Si layer thickness of (**a**) 6 nm, (**b**) 4 nm, and (**c**) 2 nm. Red dashed line highlights the TO_2_ peak of crystalline silicon (c-Si).

Raman spectroscopy was used to analyze the presence of Si-ncs in the TA SiO_2_/Si MLs, as shown in Figure 3. The deconvolution of the Raman spectra shows bands at 310 cm^−1^, 440 cm^−1^, and 480 cm^−1^ that are attributed to the longitudinal acoustic (LA), longitudinal optical (LO), and transverse optical 1 (TO_1_) vibrational optical modes. These peaks suggest that some of the excess silicon in these TA SiO_2_/Si MLs is in an amorphous nature [49], mostly for the MLs with thinner Si layers (ML64 and ML62). Then, the Si diffusion in ML64 and ML62 produces the possible formation of amorphous Si nanoclusters and the presence of a larger quantity of defects like Si-dangling bonds.

A band around 509 cm^−1^ labeled as “GB” is observed in all Raman spectra. Such a band has also been reported by Wei et al. [50], Cheng et al. [51], and Narasimha et al. [49], and it is attributed to the grain boundary (GB) interface between Si-ncs or nanoclusters and the SiO_x_ matrix [50,51]. At the same time, the contribution of the optical transverse vibrational mode 2 (TO_2_), corresponding to the c-Si signal, is also observed. The TO_2_ band is observed at 517.7 cm^−1^ for ML66, while for both ML64 and ML62, it is placed at 518.7 cm^−1^. These TO_2_ bands are red-shifted compared to c-Si (520.9 cm^−1^), and this is attributed to phonon confinement effects and forms the basis for Si-ncs size estimation [49]. Assuming spherical Si-ncs, the intensity of the Raman spectrum according to the phonon confinement model is given by the following [49,50,51,52,53]:

(1) $$Ic\left(w\right)=Bc\;\int_{0}^{1}\frac{\exp\left(-\frac{q^{2}L^{2}}{4}\right)4\pi q^{2}}{{\left[\omega \;-\;\omega (q\right)]}^{2}+{\left(\frac{\Gamma c}{2}\right)}^{2}}$$

where *Bc* is a constant, *L* = *d/a*_0_, *d* is the diameter of the Si-nc, *a*_0_ = 0.543 nm is the lattice constant of c-Si, *Γ_C_* = 3 cm^−1^ is the Raman linewidth of c-Si at room temperature T = 300 K, *q* is the phonon wave vector expressed in 2*π/a*_0_ units, and *ω (q)* is the optical phonon scattering ratio. For silicon, the dispersion relation can be approximated by the dependence *ω* (*q*) = *ω_C_* (1 − 0.18*q*^2^), where *ω_C_* = 520.9 cm^−1^ is the optical phonon frequency of c-Si at the *Γ* point of the Brillouin zone. From the c-Si shift frequency (TO_2_) calculated with Equation (1), the diameter of the Si-ncs embedded in MLs was estimated and reported in Table 1. For ML66, the Si-NC size is around 5.5 ± 0.5 nm, which is close to the thickness of the Si layers in ML (~6 nm). However, when the thickness of the Si layer decreases, the continuity of the Si layers is lost due to the high Si diffusion coefficient from the Si layers, allowing a Si-ncs size of up to 8.5 nm for ML-64 and ML-62. This Si-ncs size, larger than the thickness of the Si layer, in the MLs, is predicted from the observation of the Si diffusion behavior in the depth profile, as observed in Figure 1e,f.

**Figure 4:**
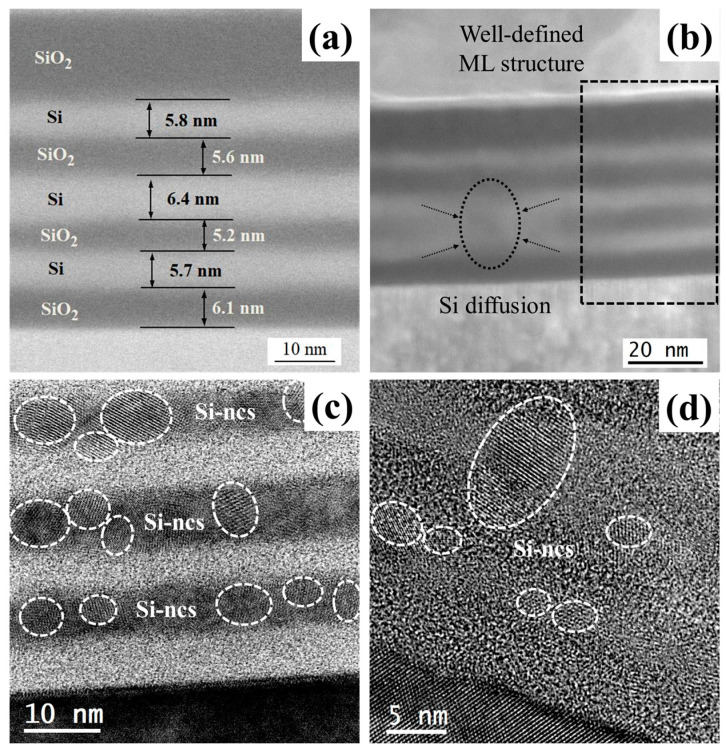
TEM micrographs of (**a**) ML-66 with a defined structure, (**b**) ML-62 with Si diffusion after thermal annealing (dotted oval and arrows indicate the discontinuity of the ML structure), (**c**) Si-ncs embedded in the ML-66 structure, and (**d**) larger Si-ncs caused by Si diffusion in ML-62.

TEM analyses were performed for ML-66 and ML-62, after thermal annealing, to corroborate the Si-ncs formation and the structure of the ML, as shown in Figure 4a,b, respectively.

As observed in Figure 4a, ML-66 exhibits a well-defined ML structure with Si and SiO_2_ layers alternating sequentially with estimated thicknesses of 6.0 ± 0.3 and 5.7 ± 0.4 nm, respectively, achieving the control of the ncs-Si growth. However, as the thickness of the Si layers decreases, as in ML-62 shown in Figure 4b, discontinuous regions are obtained where it is possible to appreciate the diffusion process of the neighboring Si layers, resulting in the partial loss of the ML structure. In fact, the dotted circle indicates a thicker Si layer because of the Si diffusion and zones where the ML retains the original design (dashed rectangle). As a result of this structural change, the possibility of obtaining larger Si-ncs increases, as estimated by XPS and Raman analysis. This information was corroborated by analyzing the TEM images (Figure 4c,d), where the presence of Si-ncs was observed in both structures. However, as previously determined, sample ML-66 showed good control with Si-ncs of around 5.5 ± 1.2 nm in the Si layers, while sample ML-62, where diffusion was observed, presented larger Si-ncs with a size of up to 9.2 nm (Figure 4d).

**Figure 5:**
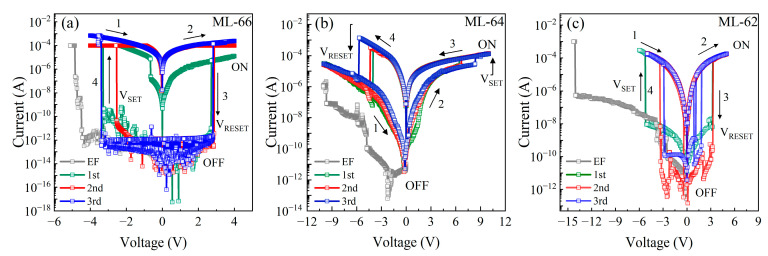
I–V curves of SiO_2_/Si MLs with the Si layer thickness of (**a**) 6 nm, (**b**) 4 nm, and (**c**) 2 nm. The numbered arrows indicate the direction of the voltage sweeps applied to the devices during each cycle, as well as the turn-on (V_SET_) and turn-off (V_RESET_) voltages.

The electrical behavior of these ML devices was studied by I–V measurements. Figure 5 shows the I–V curves of the thermally annealed ML-66 (a), ML-64 (b), and ML-62 (c) for three cycles of operation. All devices exhibit an electroforming process (EF; the initial transition from an insulating state to a conducting state) for the first I–V measurement in a negative voltage sweep (gray line). Devices with ML-66 (Figure 5a) show a HRS with a current value of about 6.3 × 10^−12^ A (OFF) for low voltages, and as the voltage increases beyond −4.5 V, three SET transitions with current levels of about 6.3 × 10^−10^, 2.4 × 10^−8^, and 1.0 × 10^−4^ A are observed to obtain the LRS (ON) state. It is evident that these three intermediate states agree with the number of Si-ncs layers. As the thickness of the Si layer decreases from 6 (ML66) to 4 (ML64) and 2 nm (ML62) (Figure 5b,c), the voltage required to obtain the EF process increases from −4.5 V up to −14.1 V, and it does not exhibit the staircase-like current behavior. Moreover, these devices present a higher current value for HRS of about 5.4 × 10^−7^ A (at V < −14.0 V), which can be related to a higher excess of Si that increases the conductivity of the devices. This behavior would be linked to the diffusion of this element to other regions by the collapse of the multilayer structure, as observed in the XPS analysis and TEM micrographs.

After the EF process, hysteresis in I–V curves is evident in both polarizations. For the ML-66 device, a voltage sweep was applied from −4 V to 0 V (1), where a high carrier flow is observed, keeping the device in the ON state. The device remains at LRS (ON) in a new measurement (at reverse bias) from 0 V to 4 V (2). However, upon sweeping from 4 V to 0 V (3), an abrupt current drop to 5.2 × 10^−12^ A (RESET process) was observed at 2.8 ± 0.1 V. A new voltage sweep from 0 V to −4 V (4) again shows the RS from HRS to LRS at −3.1 ± 0.5 V (SET process). For ML-64 (Figure 5b), after the EF process, a returning voltage sweep (1) from −10 V to 0 V shows a slight increase in current, but it can be considered as an OFF state. A new measurement from 0 V to 10 V (2) shows that the device switches from HRS to LRS (ON) at about 7.1 ± 1.1 V. The LRS (ON state) is kept for a sweep voltage from 10 to 0 V (3). A new voltage sweep at forward bias (4) shows the RESET process by an abrupt current drop from 6.6 × 10^−4^ to 1.0 × 10^−6^ A at about −4.7 ± 0.9 V. For ML-62 (Figure 5c), after the EF process, a first (1) and second voltage sweep (2) applied from −6V to 0 V and 0 V to 6 V, respectively, show a high current level of up to 4.3 × 10^−5^ A, indicating that the device is at ON state (LRS). However, upon returning the voltage sweep from 6 V to 0 V (3), the device exhibits an abrupt current drop to 1.9 × 10^−9^ A at ~2.1 ± 1.3 V. Finally, a new voltage sweep from 0 V to −6V (4) shows the new RS from HRS to LRS (SET process). The current values observed in the devices during the operating cycles are even lower than those reported in the literature for silicon oxide dielectric matrices [54,55].

As observed in Figure 5, the SET/RESET voltages ranged from −3.1 ± 0.5 V/2.8 ± 0.1 V to −3.8 ± 1.2 V/2.1 ± 1.3 V when the thickness of the silicon layer was reduced from 6 (ML-66) to 2 (ML-62) nm, respectively. ML-64 showed the highest SET/RESET voltage values of 7.1 ± 1.1 V/−4.7 ± 0.9 V, as shown in Table 2. On the other hand, the LRS/HRS ratio (ON ↑ for SET / OFF ↓ for RESET) also decreased from about 1.6 × 10^7^ for ML66 to 3.0 and 1.8 × 10^4^ for ML64 and ML62, respectively. The best LRS/HRS ratio of about 1.6 × 10^7^ obtained for ML-66 is higher than reported by other reports on devices based on SiO_x_ layers, where values between 10^5^ and 10^4^ have been obtained [31,32].

**Figure 6:**
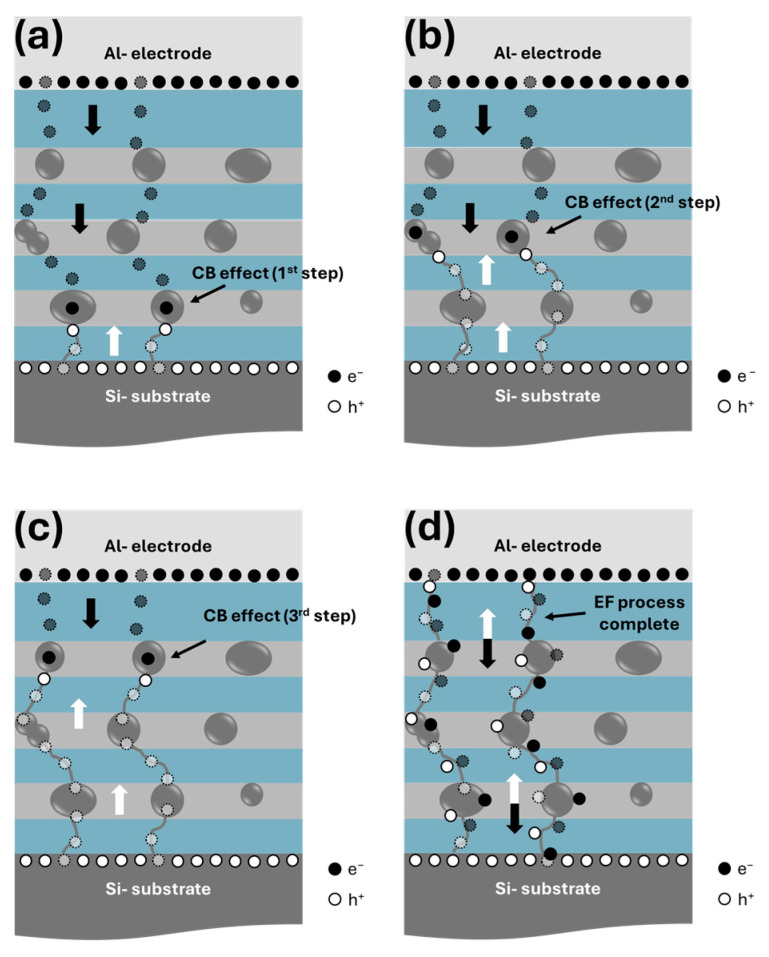
Schematic diagram of Coulombic blocking effect by trapped charge (e^−^) in MOS structure with SiO_2_/Si MLs with the Si layer thickness of 6 nm: (**a**) 1st step, (**b**) 2nd step, (**c**) 3rd step, and (**d**) electroforming (EF) process complete. The black and white arrows indicate the direction of flow of h^+^ and e^−^, respectively, while the dotted circles indicate their trajectory.

As observed in Figure 5a, the EF process obtained in ML-66 exhibited a staircase-like behavior. It is necessary to understand this phenomenon to relate it to the physics of the RS process. The ML-66 is composed of three layers of Si-ncs (~5.5 nm in size) separated by SiO_x_ layers as thin as 6 nm, which allows carrier tunneling between Si-ncs. The SiO_x_ layers contain defects, as evidenced by the high amount of Si oxidation states that allow oxygen ions to move. These SiO_x_ layers generate a lower electrical resistance, if compared to SiO_2_, between Si-ncs. Therefore, the size and position of some Si-ncs in MLs cause preferential CFs connecting the top electrode with the Si substrate. During the first voltage sweep, when forward bias is applied (−V), holes (h^+^, white circles) accumulate on the substrate surface (Si/ML interface), while electrons (e^−^, black circles) accumulate on the top electrode. These charges, either h^+^ or e^−^, tunnel into the matrix through CFs, which are formed by the mobility of oxygen ions when an electric field is applied, causing the breakdown of Si-O bonds and generating the formation of Si-Si bonds that allow the carrier flow. Charges near the Si-ncs can become trapped in them. If charge trapping occurs, the CF is deactivated due to the Coulombic blocking (CB) effect because the trapped charge (e^−^) creates a strong Coulomb repulsion that prevents other charges from flowing, as schematized in Figure 6a. Similarly, it could explain the transport mechanism for the staircase-like behavior observed in our I–V curves during the EF process (Figure 5a). As the negative voltage is increased, band bending is even more evident, so more h^+^ and e^−^ accumulate at the SiO_x_/Si and Al/SiO_x_ interface, respectively. Finally, as the voltage is increased, the charge trapped in the ncs-Si is forced to tunnel and connect with other particles (2nd and 3rd steps, Figure 6b and Figure 6c, respectively) up to the conductive path, resulting in a jump in electric current (Figure 6d). This behavior describes the initial formation of the CFs through the dielectric matrix, producing the first change from a HRS to a LRS.

This electrical behavior only occurs in the initial stage of the devices (pristine devices); once the preferential CF is complete, this phenomenon does not occur again. However, for ML-64 and ML-62, due to discontinuities in the ML structure, the stepped EF effect could not be observed, generating an increase in operating voltages. Once the conductive filaments have been established, during the RESET cycle, applying an opposite electric field promotes the recombination of oxygen ions, causing partial rupture of the conductive filament. When the process is stable and repetitive, the recovery/annihilation of the conductive filament requires less energy to form/break, which explains the reduction in operating voltages observed in the I–V curves in Figure 5. One possible alternative to avoid the loss of ML structure observed in ML-64 and ML-62 after TA is the application of a rapid thermal annealing process (RTA) to reduce the prolonged time of thermal annealing. This RTA-based step process is compatible with CMOS technology, and it has been reported for the formation of Si-ncs [56]; therefore, it will be explored in future works for RS devices.

## 4. Conclusions

SiO_2_/Si multilayer devices were successfully manufactured as resistive memories. It was possible to determine that RS performance is linked to their structural properties. Devices ML-62 and ML-64 exhibited erratic RS behavior due to a structural discontinuity of Si and SiO_2_ layers. The operating voltages in these devices increased by up to 300%, reaching values close to −9 V, compared to ML-66, whose structure remained intact, and which exhibited an operating voltage of around ±3 V and an ON/OFF ratio of up to seven orders of magnitude. Although the Si diffusion does not represent the total loss of the original structure, it modifies the electrical behavior and performance of the devices, demonstrating the importance of preserving the original ML structure for better performance of RS devices.

## Figures and Tables

**Table 1:** Thickness (Th) of SiO_2_ and Si layers in MLs, TO_2_ Raman peak position, and diameter (d) of the Si-nc.

Sample	Th (nm)		TO_2_ (cm^−1^)	d (nm)
	SiO_2_	Si		
ML-66	6	6	517.7	~6.0
ML-64		4	518.7	~8.5
ML-62		2	518.7	~8.5

**Table 2:** Electrical resistive switching parameters for ML-66, ML-64, and ML-62 devices.

Device	SET Voltage	HRS SET	LRS SET	LRS/HRS Ratio	RESET Voltage	HRS RESET	LRS RESET	LRS/HRS Ratio
	V	A		ON ↑	V	A		OFF ↓
ML-66	−3.1 ± 0.5	1.6 × 10^−11^	2.7 × 10^−4^	1.6 × 10^7^	2.8 ± 0.1	5.2 × 10^−12^	8.2 × 10^−5^	1.6 × 10^7^
ML-64	7.1 ± 1.1	2.5 × 10^−5^	7.4 × 10^−5^	3.0	−4.7 ± 0.9	1.0 × 10^−6^	6.6 × 10^−4^	6.7 × 10^2^
ML-62	−3.8 ± 1.2	5.5 × 10^−9^	9.7 × 10^−5^	1.8 × 10^4^	2.1 ± 1.3	1.9 × 10^−9^	4.3 × 10^−5^	2.2 × 10^4^

## Data Availability

The data that support the findings of this study are available from the corresponding author upon request.
